# Patient-reported outcomes for diabetes and hypertension care in low- and middle-income countries: A scoping review

**DOI:** 10.1371/journal.pone.0245269

**Published:** 2021-01-15

**Authors:** Sarah Masyuko, Carrie J. Ngongo, Carole Smith, Rachel Nugent

**Affiliations:** 1 RTI International, Seattle, Washington, United States of America; 2 Department of Global Health, University of Washington, Seattle, Washington, United States of America; 3 Ministry of Health, Nairobi, Kenya; 4 Department of Neurology, University of Washington, Seattle, Washington, United States of America; Universidade de Mogi das Cruzes, BRAZIL

## Abstract

**Introduction:**

Patient-reported outcome measures (PROMs) assess patients’ perspectives on their health status, providing opportunities to improve the quality of care. While PROMs are increasingly used in high-income settings, limited data are available on PROMs use for diabetes and hypertension in low-and middle-income countries (LMICs). This scoping review aimed to determine how PROMs are employed for diabetes and hypertension care in LMICs.

**Methods:**

We searched PubMed, EMBASE, and ClinicalTrials.gov for English-language studies published between August 2009 and August 2019 that measured at least one PROM related to diabetes or hypertension in LMICs. Full texts of included studies were examined to assess study characteristics, target population, outcome focus, PROMs used, and methods for data collection and reporting.

**Results:**

Sixty-eight studies met the inclusion criteria and reported on PROMs for people diagnosed with hypertension and/or diabetes and receiving care in health facilities. Thirty-nine (57%) reported on upper-middle-income countries, 19 (28%) reported on lower-middle-income countries, 4 (6%) reported on low-income countries, and 6 (9%) were multi-country. Most focused on diabetes (60/68, 88%), while 4 studies focused on hypertension and 4 focused on diabetes/hypertension comorbidity. Outcomes of interest varied; most common were glycemic or blood pressure control (38), health literacy and treatment adherence (27), and acute complications (22). Collectively the studies deployed 55 unique tools to measure patient outcomes. Most common were the Morisky Medication Adherence Scale (7) and EuroQoL-5D-3L (7).

**Conclusion:**

PROMs are deployed in LMICs around the world, with greatest reported use in LMICs with an upper-middle-income classification. Diabetes PROMs were more widely deployed in LMICs than hypertension PROMs, suggesting an opportunity to adapt PROMs for hypertension. Future research focusing on standardization and simplification could improve future comparability and adaptability across LMIC contexts. Incorporation into national health information systems would best establish PROMs as a means to reveal the effectiveness of person-centered diabetes and hypertension care.

## Background

Non-communicable diseases (NCDs) account for 71% of global deaths [1]. Rapid societal change is driving dramatic NCD growth particularly in low-and middle-income countries (LMICs), posing a challenge to health systems [2]. The estimated global prevalence of diabetes is 9.3%, and this is projected to rise to 10.2% by 2030 [3]. Nearly 4 out of 5 people living with diabetes (79%) live in LMICs, although the prevalence of diabetes is higher in high-income countries (10.4%) and middle-income countries (9.5%) than in low-income countries (4.0%) [3]. Hypertension is also a growing concern, affecting an estimated 31.1% of adults [4]. The age-standardized prevalence of hypertension is rising in LMICs, even as it decreases in high-income countries [4]. Over 85% of “premature” NCD deaths before age 70 occur in LMICs, revealing inadequate detection, screening, and treatment. In both high- and middle-income countries, poor people are most at risk [3, 5].

Chronic diseases require person-centeredness and consistent, holistic care to ensure good outcomes [6, 7]. Such care can be difficult to provide through LMIC health systems built to respond to acute emergencies and infectious diseases, which may struggle to provide continuity of care. As the burden of NCDs such as diabetes and hypertension grows in LMICs, a critical question is how to continually measure and improve the quality of people’s care.

A patient-reported outcome is defined as any report of the status of a patient’s health condition that comes directly from the patient, without interpretation of the patient’s response by a clinician or anyone else. Patient-reported outcome measures (PROMs) describe patients’ perceptions of the benefits that they receive from the health system, including patient views on health outcomes and the quality of services received [8–10]. PROMs ascertain the patient’s view of their symptoms, functional status, and health-related quality of life [8]. Usually consisting of questionnaires for patient completion or response, PROMs transform subjective data to objective data using validated tools, providing a comprehensive assessment of patient health status. PROMs can be paired with patient-reported experience measures (PREMs), which are questionnaires that document patient experience with the health system [11].

PROMs and PREMs are increasingly used by clinicians and hospitals to guide clinical decision-making and for public reporting of health system performance [12]. PROMs are currently being used in high-income countries in the movement toward pay-for-performance or value-based care, where health systems, hospitals, and providers are paid for outcomes that they achieve, such as tobacco cessation or glycemic control. Some countries have successfully included PROMs in their national registries, including Sweden, Australia, and New Zealand [12]. The World Economic Forum developed a framework to guide implementation of value-based care in well-resourced settings that includes collection of select PROMs [13]. Little is known, however, about the use of PROMs in LMICs given a paucity of data. This scoping review aims to fill this gap in knowledge. The objective of this scoping review is to determine whether PROMs for hypertension and diabetes patients are being applied in LMICs. If so, how?

## Materials and methods

This review followed the Preferred Reporting Items for Systematic Reviews and Meta Analyses—Extension for Scoping Reviews (PRISMA-ScR) guidelines [14]. The protocol is available upon request from the corresponding author.

### Eligibility criteria

We included studies conducted in LMICs that were: (1) in English, (2) published in a peer-reviewed journal in the 10 years before August 8, 2019 to reflect the period when PROMs in LMICs began to appear in the published literature, and (3) reported use of at least one PROM or a single financial PREM related to hypertension, diabetes, or both (Table 1). Among LMICs, country income levels were categorized as low, lower-middle or upper-middle income as defined by the World Bank for the year 2019 [15]. The review searched for quantitative and qualitative outcomes in the standard PROMs sets for diabetes and hypertension from the International Consortium of Health Outcomes Measurement and specified quality of life and patient satisfaction as separate outcomes (Table 1) [16, 17] Given that financial barriers significantly constrain healthcare utilization in many LMICs, we included one financial PREM that reported on economic accessibility as part of this review. The review included studies that reported on preferences, acceptability or feasibility of using PROMs. Values and preferences studies were included only if they presented primary data examining the values and preferences of potential beneficiaries, communities, providers, and stakeholders. We excluded letters, editorials, reviews, and abstract-only publications. In addition, we excluded studies that (1) did not include at least one LMIC, (2) were conducted at population level without reference to health facilities, (3) interviewed caregivers and family members, but not patients, or (4) focused on interventions that have only an indirect impact on diabetes or hypertension.

**Table 1:** Patient reported outcome measures of interest.

Outcome	Details
**PROMS for Hypertension and Diabetes**	
Disease control	• Blood pressure control among patients with hypertension • Glycemic control (Hemoglobin A1c (HbA1c) or fasting blood glucose) among patients with diabetes
Health literacy and treatment adherence	• Beliefs about medication • Adherence to medication
Acute complications	• Ketoacidosis and Hyperosmolar Hyperglycemic Syndrome • Hypoglycemia • Acute cardiovascular events (stroke and myocardial infarction) • Lower limb amputation
Chronic complications	• Chronic complications related to vision, autonomic neuropathy, peripheral neuropathy, Charcot’s foot, lower limb ulcers, peripheral artery disease, ischemic heart disease, chronic heart failure, chronic kidney disease and dialysis, cerebrovascular disease, periodontal health, erectile dysfunction (males) and lipodystrophy (for persons on injectable insulin or non-insulin therapies)
Quality of life	• Pain or discomfort • Anxiety or depression • Difficulty functioning (walking, washing or dressing oneself, doing usual activities
Burden of care	• Access to care • Access to medication • Pill burden
Health services	• Hospitalization • Emergency room utilization
Self-care efficacy	• Patient’s confidence in their own pain and symptom management, information management, medication taking, home/self-monitoring of blood pressure or blood sugar, diet, exercise
Psychological wellbeing, stress, depression	
Patient satisfaction	
**Access PREM**	
Economic accessibility	• Health insurance coverage • Out-of-pocket payments for services related to diabetic or hypertensive care • Inability to access recommended care due to inability to pay

### Search strategy

The review searched PubMed, EMBASE, and ClinicalTrials.gov for randomized controlled trials through 8 August 2019. The search included three components: (1) a PROMs component and (2) a disease component (diabetes and/or hypertension) and (3) a list of LMICs. Search terms were customized for each electronic database. The full strategy is available as S1 Appendix.

### Data analysis

#### Screening and data extraction

We used Covidence (Veritas Health Innovation Ltd, Melbourne, Australia) to manage search results and determine review eligibility. We first merged search results from each database and removed duplicate citations. Two reviewers independently screened titles and abstracts of all search results, retrieved full-text articles for the abstracts that received two votes for inclusion, and independently screened the full texts. Studies identified from ClinicalTrials.gov were identified as potentially eligible following title and abstract review. Associated full-text articles were included if available. Conflicts were resolved through reviewer discussion. A senior reviewer (CN) verified eligibility for inclusion during the full-text review only. The reviewers (CS, SM) extracted data from included articles into Microsoft Excel (Microsoft Corporation, Redmond, WA).

#### Intervention categories and stratification

PROMs and the financial PREM were divided by disease focus: 1) diabetes, 2) hypertension, and 3) both diabetes and hypertension. Articles were further sub-divided by:

1. Location (World Bank income groupings, World Health Organization (WHO) regions [15, 18]
2. Study population
3. Study design
4. Level of health facility
5. Tools used, status of tool/questionnaire validation and domains measured
6. Administration (clinician, external body, self-administered)
7. Method of data collection (electronic or manual)
8. Frequency of evaluation
9. Technology use (digital health, telemedicine)
10. Intended use (financial or non-financial incentives, clinical decision-making, quality improvement)

## Results

### Search results

**Fig 1:**
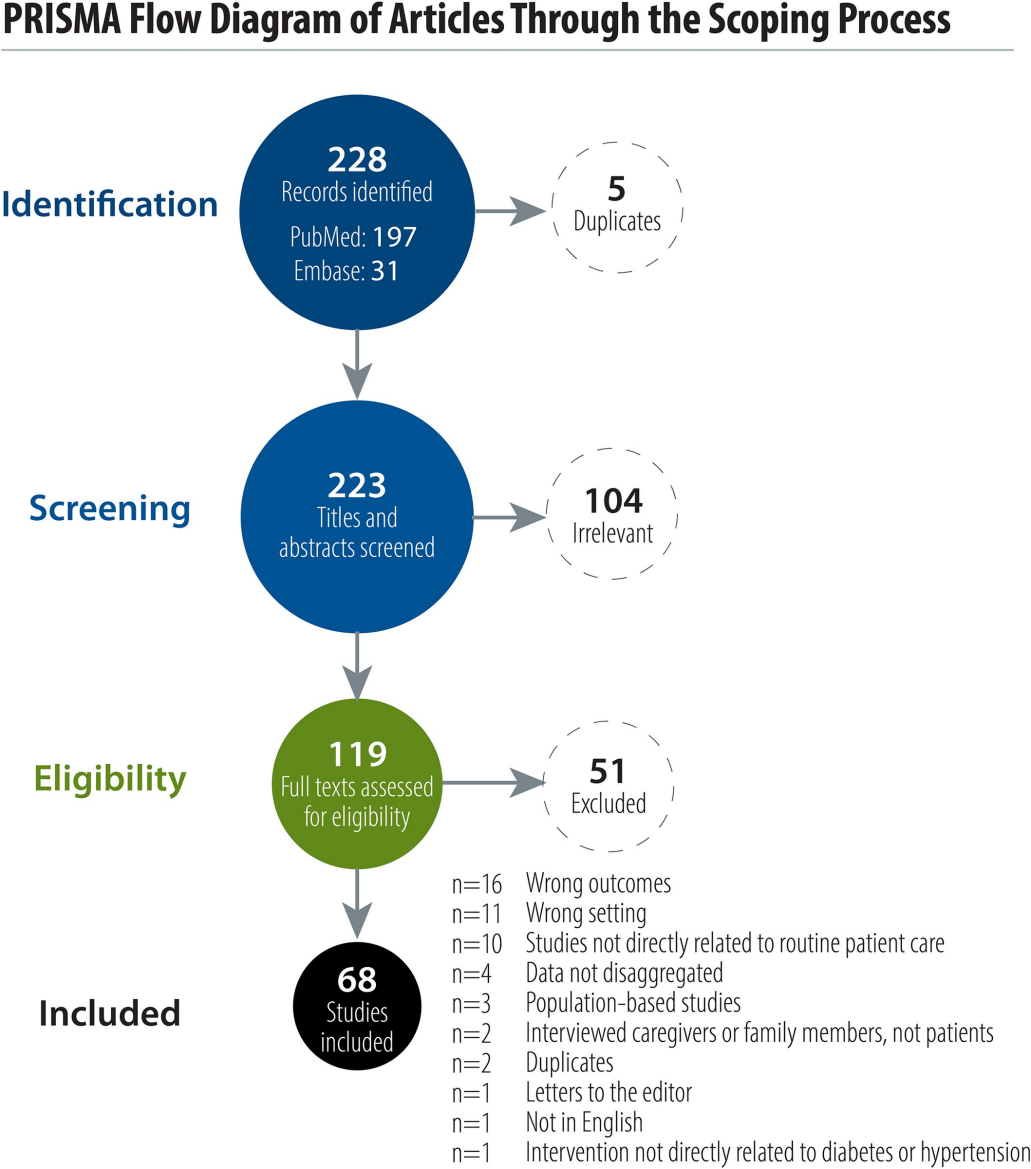
PRISMA flow diagram of articles through the scoping process.

Our search identified 197 studies from PubMed and 31 studies from Embase that met our study criteria. None of the studies identified through ClinicalTrials.gov met the study criteria. Out of the 228 identified articles reporting PROMs in LMICs, 5 duplicate studies were removed. After screening of titles and abstracts, 119 studies proceeded to full-text review and 68 studies were eligible and included in this review (Fig 1).

### Study findings

#### Where have PROMs been collected in LMICs?

**Fig 2:**
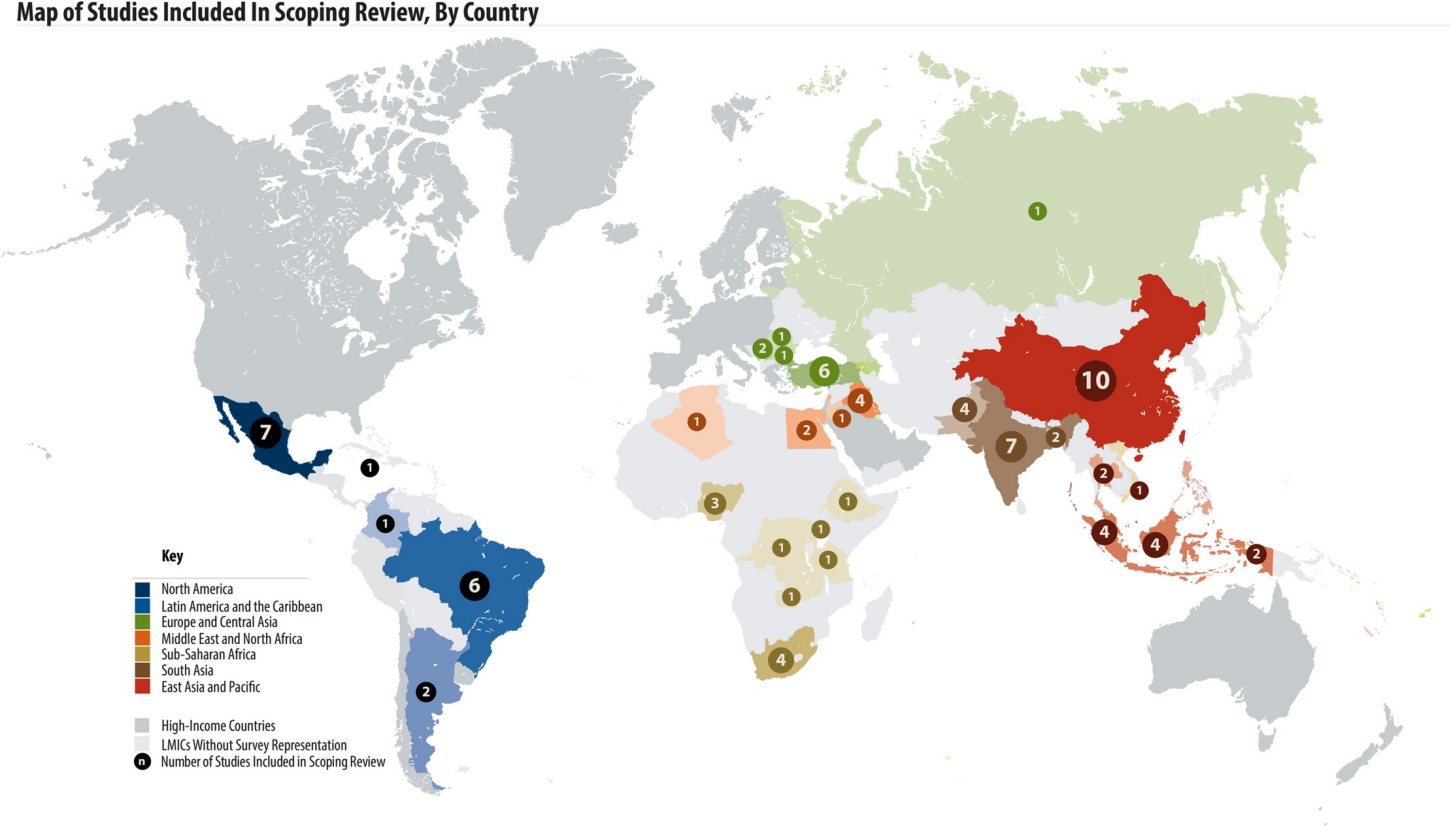
Map of studies included in scoping review, by country.

PROMs have been used in LMICs all over the world. Thirty-one LMIC countries from all six WHO regions are represented in the 68 studies included in this review (Fig 2). Among included studies, 39/68 (57%) were from upper-middle-income countries, followed by 19/68 (28%) from lower-middle income countries, and 4/68 (6%) from low-income countries. The region of the Americas reported the most studies (n = 13, 19%). Six studies were multi-country studies: 4 including countries from multiple WHO regions and 5 including countries with multiple World Bank income groupings. Out of the 68 studies, 60 (88%) reported patient reported outcomes on diabetes, 4 (6%) reported on hypertension and 4 (6%) reported on both diabetes and hypertension.

The included studies were published between 2010 and 2019, with 45 studies (66%) published between 2016 and 2019. The majority were cross-sectional studies (n = 35, 51%), followed by prospective cohort studies (n = 16, 23%), both prospective and retrospective cohort studies (n = 7, 10%), qualitative studies (n = 4, 6%), descriptive studies (n = 3, 4%), randomized clinical trials (n = 2, 3%) and case-control studies (n = 1, 1%). Baseline characteristics of the included articles are shown in Table 2.

**Table 2:** Baseline characteristics of included studies.

	Diabetes	Hypertension	Diabetes and Hypertension	Total
	(n = 60)	(n = 4)	(n = 4)	(n = 68)
	n (%)	n (%)	n (%)	n (%)
**Years of publication**				
2010–2015	20 (33)	2 (50)	2 (50)	24 (35)
2016–2019	40 (67)	2 (50)	2 (50)	44 (65)
**Countries involved**				
Single country	54 (90)	4 (100)	4 (100)	62 (91)
Multi-country	6 (10)	0 (0)	0 (0)	6 (9)
**World Bank income grouping**				
Low-income	4 (7)	0 (0)	0 (0)	4 (6)
Lower-middle	18 (30)	1 (25)	0 (0)	19 (28)
Upper-middle	32 (53)	3 (75)	4 (100)	39 (57)
Multi-income	6 (10)	0 (0)	0 (0)	6 (9)
**WHO regions**				
Africa	10 (17)	1 (20)	0 (0)	11 (16)
Americas	9 (15)	0 (0)	4 (100)	13 (19)
Europe	4 (7)	1 (20)	0 (0)	5 (7)
Mediterranean	10 (17)	1 (20)	0 (0)	11 (16)
South East Asia	11 (18)	0 (0)	0 (0)	11 (16)
Western Pacific	11 (18)	1 (20)	0 (0)	12 (18)
Multi-region	5 (8)	0 (0)	0 (0)	5 (7)
**Study design**				
Cross-sectional	34 (57)	0 (0)	1 (25)	35 (51)
Prospective cohort	11 (18)	3 (75)	2 (50)	16 (23)
Both prospective and retrospective cohort	7 (12)	0 (0)	0 (0)	7 (10)
Qualitative	3 (5)	1 (25)	0 (0)	4 (6)
Randomized clinical trial	2 (3)	0 (0)	0 (0)	2 (3)
Descriptive	3 (8)	0 (0)	0 (0)	3 (4)
Case-control	0 (0)	0 (0)	1 (25)	1 (1)

### How were studies conducted? Which key domains were measured?

#### Diabetes mellitus

*Study population*. Participants with established diabetes were reported in 54 studies (90%). Thirty-two studies (53%) focused on Type 2 diabetes, 3 studies (5%) reported on Type 1 diabetes, and 21 studies (35%) reported on both Type 1 and 2 diabetes. Three studies (5%) included adolescents (>12 years), while the remaining 57/60 (95%) focused on adults older than 18 years. Two studies (3%) enrolled older participants (>55 years). Participants were mainly drawn from tertiary hospitals (29/60, 48%). Most studies (41/60, 68%) had sample sizes of 500 or fewer. The inclusion criteria for 34 studies (57%) included the treatment regimen, such as insulin, diet/exercise, oral hypoglycemic agents, or combination therapies. In addition to the inclusion criteria, 15 studies (25%) discussed treatment approaches for responding patients.

*Patient-reported outcomes*. Twenty-seven studies (45%) provided outcome data provided by patients only, while 33 studies (55%) described patient-reported outcomes that had been validated by clinician data. A majority of studies (49/60, 82%) reported on three or fewer patient-reported outcomes. The most commonly reported outcome was glycemic control (36 studies, 60%), followed by health literacy and treatment adherence (23 studies, 38%), acute complications (21 studies, 35%), chronic complications (19 studies, 32%), quality of life (17 studies, 28%), economic accessibility (15 studies, 25%), psychological wellbeing, diabetic stress and depression (13 studies, 22%), patient satisfaction (9 studies, 15%), self-care efficacy (6 studies, 10%), and health services (5 studies, 8%).

*Data collection/reporting*. Two-thirds of studies (40/60, 67%) assessed outcomes once. Frequency of follow-up varied for the remaining 20 studies (33%), ranging from one week to two years. All 60 studies were conducted as stand-alone surveys; only one study used routinely collected patient-reported outcomes from existing records. Questionnaires were administered by study staff with chart review in 23 studies (38%) and by clinicians in 4 studies (7%). Questionnaires were self-administered by the patients in 13 studies (22%) while the remaining 20 studies did not specify (33%).

#### Hypertension

*Study population*. All four hypertension PROMs studies targeted adult populations with an established diagnosis of hypertension. Two studies targeted adults ≥18yrs, one study targeted adults ≥55, and one study targeted adults ≥65 yrs. Inclusion criteria included the use of anti-hypertensive medication in 2/4 studies. PROMs were collected at a single time point in either primary (2/4) or tertiary (2/4) health care facilities.

*Patient-reported outcomes*. Three of the four studies of hypertension patient outcomes collected data from patients only, while one study collected data from patients and validated with clinician data. Health literacy and treatment adherence was the most frequently reported focus, reported in 3/4 studies. The following outcomes each appeared in one study: quality of life, burden of care, patient satisfaction, economic accessibility, affordability of transportation costs, and health services as measured by prior hospitalization/admission.

*Data collection/reporting*. All four studies reported on patient reported outcomes as a primary outcome and used stand-alone surveys. One study reported data collected by a staff-administered survey, one reported on focus group discussions, and two did not specify their data collection method.

#### Diabetes and hypertension

*Study population*. All four diabetes and hypertension PROMs studies reported on adult populations. Targeted populations included adults ≥18 years with two studies focusing on adults ≥50 years. The studies focused on patients with an already-established diagnosis for hypertension and/or diabetes. Three of four studies measured use of medication. Two studies collected PROMs at a single time point, one study collected PROMs at baseline and six months, while one study collected PROMs at baseline and every three months for two years.

*Patient-reported outcomes*. Patient-reported outcomes were validated with clinical data in three of the four studies. Health literacy and treatment adherence was the most frequently reported outcome (3 studies) followed by diabetic/hypertensive chronic complications (2 studies), glycemic control (2 studies), blood pressure control (2 studies), diabetic/hypertensive acute events (2 studies), economic accessibility (2 studies), and patient satisfaction (1 study).

*Data collection/reporting*. All four studies collected patient reported outcomes as a primary outcome and the surveys were administered by study staff. One study used face-to-face interviews. The survey design from the other three studies was unspecified.

### Summaries of the PROMs used and what they measured

**Fig 3:**
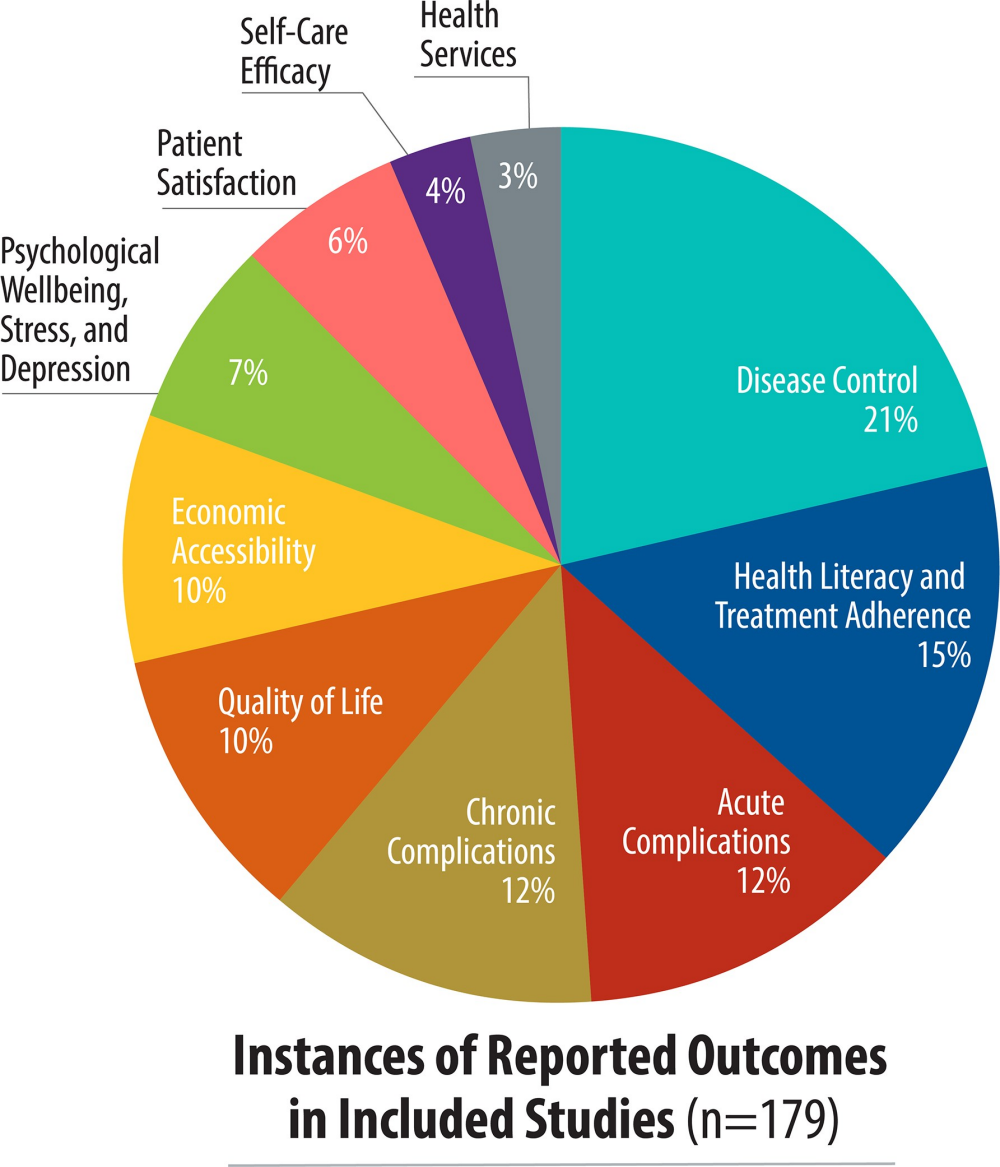
Study focus, by patient-reported outcome.

**Fig 4:**
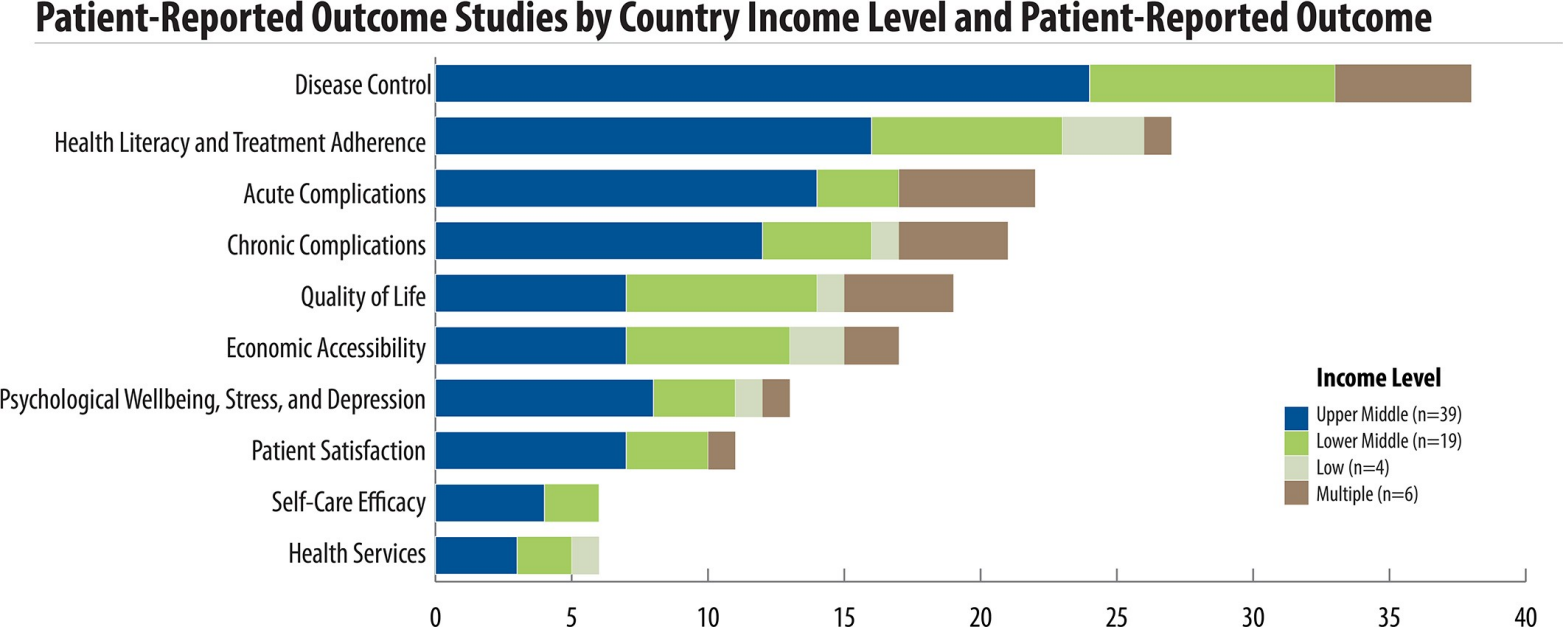
Patient-reported outcome studies by country income level.

There was great variation on the outcomes reported (Fig 3). Overall, the five most common patient-reported outcomes were disease control (38 studies), health literacy and treatment adherence (27 studies), acute complications (22 studies), chronic complications (21 studies), and quality of life (18 studies). Health literacy and medication adherence was the most reported outcome in low-income countries as compared to disease control in lower- and upper-middle income countries. In multi-income country studies, acute complications and disease control were the most reported outcomes (Fig 4). Disease control, acute complications, patient satisfaction, and self-care efficacy outcomes were not reported in low-income countries. While disease control was the most-reported outcome, it was measured primarily with clinical data rather than a specific tool.

Studies reported a combined total of 55 unique tools to collect PROMS. Most tools focused on diabetes alone (51/55, 92%), while four tools focused on hypertension and two tools were used for both hypertension and diabetes (Table 3). One study used a single tool that incorporated various PROMs to assess multiple patient-reported outcomes. Table 3 summarizes the tools used, domains measured, and scale of studies reporting on each tool.

**Table 3:** Patient-reported outcomes and the tools used to measure each outcome.

Outcome (number of studies)	Tool	Reported uses	Specific focus of tool	Disease-specific or generic	Validated in English	Validated version used	Language, if specified	References of studies reporting the PROM
Health Literacy and Treatment Adherence [20]	Morisky Medication Adherence Scale (MMAS) [19]	7		Generic	Y	Y	Brazilian Portuguese, India (unspecified), Portuguese, Turkish, and not specified (2)	do Valle Nascimento *et al*. 2017 [20], Gomes *et al*. 2016 [21], Pirdehghan *et al*. 2016 [22], Swain *et al*. 2018 [23]; Saqlain *et al*. 2019 [24]; Wang *et al*. 2014 [25]; Baran *et al*. 2017 [26]
	Brief Diabetes Knowledge Test [27]	2		Disease-specific	Y	N	Asian, not specified (1)	Linetzky *et al*. 2016 [28]; Matsuba *et al*. 2016 [29]
	Self-Efficacy about Insulin Therapy Questionnaire (SEITQ) [30]	2		Disease-specific	Y	N	MENA languages (unspecified)	Jabbar *et al*. 2019 [31]; Jabbar *et al*. 2018 [32]
	Single Item Literacy Screener (SILS) [33]	1		Generic	Y	Not specified	Not specified	Saqlain *et al*. 2019 [24]
	RAND Improved Chronic Illness for Care Evaluation study [34]	1		Disease-specific	Y	Not specified	Not specified	Kleinman *et al*. 2017 [35]
	Centre for Adherence Support Evaluation (CASE) Adherence Index [36]	1		Generic	Y	Not specified	Not specified	Newman *et al*. 2018 [37]
	Drug Attitude Inventory (DAI-10) [38]	1		Generic	Y	Y	Urdu	Iqbal *et al*. 2017 [39]
	Insulin Specific Adherence Questionnaire	1		Disease-specific	Not available	Not available	Not specified	Linetzky *et al*. 2016 [28]
	Michigan Diabetes Knowledge Test (MDKT) [40]	1		Disease-specific	Y	Y	Urdu	Iqbal *et al*. 2017 [39]
	Modified “Show and Tell” questions (MSTQ)	1		Generic	N/A	N/A	N/A	Adisa *et al*. 2014 [41]
	Recognize, Identify, and Manage (RIM)	1		Generic	N/A	N/A	N/A	Adisa *et al*. 2014 [41]
	Modified Neuropathy Symptom Score (NSS) [42]	1	Diabetic Neuropathy	Disease-specific	Y	N	South Africa (unspecified)	Kemp *et al*. 2015 [43]
Quality of life [18]	EQ-5D/EQ-5D-3L questionnaire-VAS [44]	7		Generic	Y	Y, N	Chinese, India (unspecified), Middle East and North Africa (MENA) languages (unspecified), and Serbian	Guo *et al*. 2018 [45]; Jabbar *et al*. 2019 [31]; Jabbar *et al*. 2018 [32]; Moses *et al*. 2013 [46]; Nguyen *et al*. 2018 [47]; Shah *et al*. 2014 [48]; CvetanoviÄ‡ *et al*. 2017 [49]
	Audit of diabetes-dependent quality of life-19 (ADDQOL-19) [50]	2		Disease-specific	Y	Y, N	Kannada/English, not specified (1)	Ahammed *et al*. 2018 [51]; PrasannaKumar *et al*. 2018 [52]
	Medical Outcomes Study (MOS) Short-Form-12 (SF-12) [53]	1		Generic	Y	Y, N	Bulgarian, Greek, Dutch, Spanish, and English.	Doubova *et al*. 2013 [54]
	WHO Quality of Life Measure (WHO-QoL-Bref) [55]	2		Generic	Y	Y, not specified	Turkish; not specified (1)	Akena *et al*. 2015 [56]; Cinar *et al*. 2012 [57]
	Barthel index [58]	1	Performance in Activities of Daily Living	Generic	Y	Not specified	Not specified	Saqlain *et al*. 2019 [25]
	Diabetes-Specific Quality-of-Life Scale (DSQOLS) [59]	1		Disease-specific	Y	N	Bengali, Spanish, Egyptian, Indonesian, Filipino, Malay, South Africa (unspecified), Turkish, and Arabic	Emral *et al*. 2017 [60]
	The Well-Being Questionnaire-12 (WBQ-12)	1		Generic	Y	Y	Arabic	AbuSheikh *et al*. 2018 [61]
	Standard Gamble (SG) [62]	1		Generic	N/A	N/A	N/A	Alinia *et al*. 2017 [63]
	Time Trade-Off (TTO) [62]	1		Generic	N/A	N/A	N/A	Alinia *et al*. 2017 [63]
	Visual analogue scale (VAS) [62]	1		Generic	N/A	N/A	N/A	Alinia *et al*. 2017 [63]
Psychological wellbeing, stress, and depression [14]	Diabetes Distress Scale (DDS) [64]	3	Diabetic stress	Disease-specific	Y	Y, N, not specified	Brazilian, English, and not specified (1)	Linetzky *et al*. 2016 [28]; Matsuba *et al*. 2016 [29]; Zanchetta *et al*. 2016 [65]
	Problem Areas in Diabetes [66]	3	Emotional stress	Disease-specific	Y	Y, not specified	Chinese, Spanish, and not specified (1)	Gomez-Peralta *et al*. 2018 [67]; Guo *et al*. 2018 [45]; Kleinman *et al*. 2017 [35]
	WHO Well-Being Index 5 (WHO-5) [68]	2	Psychological well being	Generic	Y	Not specified	Chinese, not specified (1)	64, 146
								Guo *et al*. 2018 [45]; Pan *et al*. 2012 [69]
	Hamilton Depression Rating Scale [70]	1	Depression	Generic	Y	Not specified	Not specified	Gomez-Peralta *et al*. 2018 [67]
	Major Depression Inventory (MDI) [71]	1	Depression	Generic	Y	Not specified	Not specified	Hapunda *et al*. 2017 [72]
	Mini international neuropsychiatric inventory [73]	1	Depression and suicide	Generic	Y	Not specified	Not specified	Akena *et al*. 2015 [56]
	Patient Health Questionnaire-9 (PHQ-9) [74]	1	Depression	Generic	Y	Not specified	Not specified	Kowalski *et al*. 2017 [75]
	Suicide Intent Scale [76]	1	Suicide	Generic	Y	Y	Spanish	Gomez-Peralta *et al*. 2018 [67]
	Symptoms Checklist (SCL-20) [77]	1	Depression	Generic	Y	Not specified	Not specified	104
								Kowalski *et al*. 2017 [75]
Self-care efficacy [12]	Summary of Diabetes Self-Care Activities (SDSCA) [78]	4	Physical activity	Disease-specific	Y	Y, N	Chinese, Bulgarian, Greek, Malay, Dutch, Norwegian, Portuguese, Spain, English, and not specified (1)	do Valle Nascimento *et al*. 2017 [20]; Guo *et al*. 2018 [45]; Kleinman *et al*. 2017 [35]; Tan *et al*. 2011 [79]
	Diabetes Empowerment Scale-DAWN Short Form (DES-DSF) [80]	1		Generic	Y	N	Chinese	Guo *et al*. 2018 [45]
	Diabetes self-care inventory [81]	1		Disease-specific	Y	Not specified	Not specified	Hapunda *et al*. 2017 [72]
	Dietary History Questionnaire [82]	1	Dietary habits	Generic	Y	Y	Malay	Tan *et al*. 2011 [79]
	Health Behavior Questionnaire [83]	1	Physical activity	Generic	Y	Y	Turkish	Cinar *et al*. 2012 [57]
	Tooth brushing self-efficacy scale (TBSES) [84]	1	Tooth brushing	Generic	Y	N	Turkish	Cinar *et al*. 2012 [57]
	Modified Instrument to Measure Lifestyle in Diabetics (IMEVID) s [85]	1	Lifestyle	Disease-specific	Data not available	Y	Spanish	Cueto-Manzano *et al*. 2010 [86]
	Stanford Patient Education Research Center (PERC) [87]	1		Disease-specific	Y	Not specified	Not specified	Kleinman *et al*. 2017 [35]
	Exercise Self-care Agency scale (ESCA) [88]	1		Generic	Y	N	Chinese	Wang *et al*. 2014 [25]
Chronic complications [8]	International Index of Erectile Function (IIEF) [89]	2	Sexual dysfunction	Generic	Y	Not specified	Not specified (2)	Kiskac *et al*. 2015 [90]; Ziaei-Rad *et al*. 2010 [91]
	Androgen Deficiency in the Aging Male questionnaire (ADAM) [92]	1	Low testosterone	Generic	Y	Not specified	Not specified	Kemp *et al*. 2015 [43]
	Michigan Neuropathic Screening Instrument (MNSI) [93]	1	Diabetic neuropathy	Disease-specific	Y	Not specified	Not specified	Akena *et al*. 2015 [56
	Numeric Rating Scale (NRS-11) [94]	1	Pain	Generic	N	N	Urdu	Ahmed *et al*. 2018 [95]
	Staged Diabetes Management Questionnaire (SDM)	1		Disease-specific	Y	Not specified	Not specified	Rodriguez-Saldana *et al*. 2010 [96]
	The Female Sexual Function Index (FSFI) [97]	1	Sexual dysfunction	Generic	Y	Not specified	Not specified	Ziaei-Rad *et al*. 2010 [91]
	World Health Organization’s Rose Angina Questionnaire [98]	1	Intermittent claudication	Disease-specific	Y	N	South Africa (unspecified)	Kemp *et al*. 2015 [43]
Acute complications [6]	Hypoglycemia Assessment Tool (HAT)	5	Hypoglycemia	Disease-specific	N	N	Bulgarian, Croatian, Czech, Hungarian, Polish, Romanian, Russian, Serbian, Slovak, Slovenian, Spanish, Mexican, Hebrew, Lebanese, Arabic, German (2), English, Danish, Finish, Dutch, Swedish, Indian (unspecified), Malay	Emral *et al*. 2017 [60]; Hussein *et al*. 2017 [99]; Khunti *et al*. 2016 [100]; Khunti *et al*. 2017 [101]; Omar *et al*. 2018 [102]
	Staged Diabetes Management Questionnaire (SDM)	1		Disease-specific	Y	Not specified	Not specified	Rodriguez-Saldana *et al*. 2010 [96]
Patient satisfaction [7]	Patient Assessment of Chronic Illness Care (PACIC) [103]	2		Generic	Y	Y	Chinese, Portuguese	do Valle Nascimento *et al*. 2017 [20]; Guo *et al*. 2018 [45]
	Diabetes Treatment Satisfaction Questionnaire Status (DTSQs) [104]	1		Disease-specific	Y	Y	Arabic	AbuSheikh *et al*. 2018 [61]
	Interpersonal Processes of Care (IPC) [105]	1	Patient-provider relationship	Generic	Y	Not specified	Not specified	Linetzky *et al*. 2016 [28]
	Patient Satisfaction Questionnaire (PSQ-18) [105]	1		Generic	Y	Not specified	Not specified	Kleinman *et al*. 2017 [35]
	Health Care Climate Questionnaire (HCCQ)	1	Counselling	Generic	Y	N	Portuguese	do Valle Nascimento *et al*. 2017 [20]
	Fear of Intimacy with Helping Professionals scale (FIS-HP) [106]	1	Attitude/belief towards seeking help from a “human service professional.”	Generic	Y	N	Chinese	Wang *et al*. 2014 [25]
Disease control [1]	Staged Diabetes Management Questionnaire (SDM)	1		Disease-specific	Y	Not specified	Not specified	Rodriguez-Saldana *et al*. 2010 [96]

**Fig 5:**
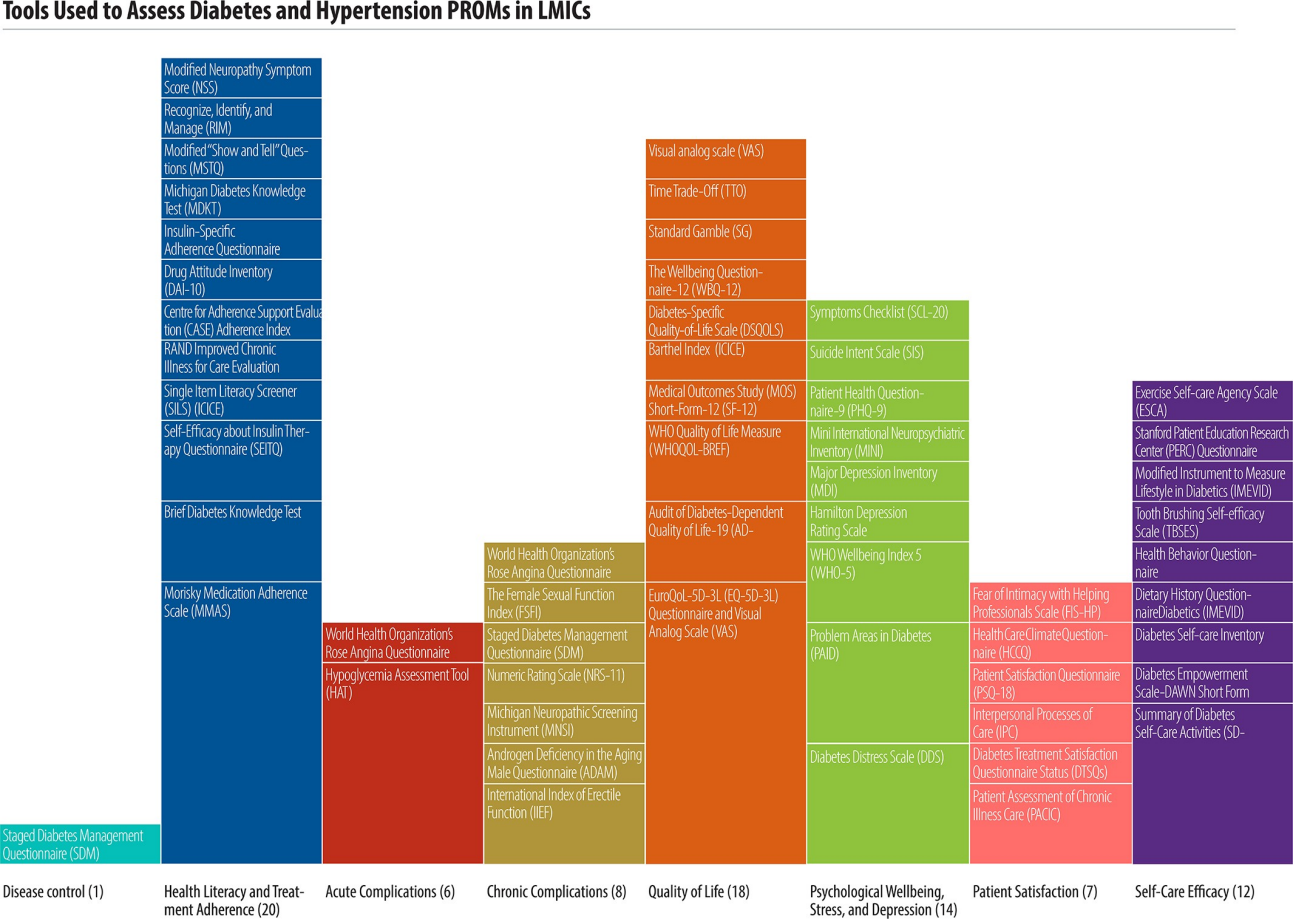
Tools used to assess diabetes and hypertension PROMs in LMICs.

*Two main types of PROMs were utilized*. generic and disease-specific. Nineteen (35%) of the 55 tools were specific to diabetes, while the remainder were generic for use across many conditions. Forty-six (84%) of the 55 tools have been validated in English; studies reported extensive translation for use in multiple other languages (Table 3). Many tools focused on health literacy and treatment adherence (20 studies), quality of life (19 studies), and psychological well-being, stress, and depression (14 studies). The most common reported tools were the Morisky Medication Adherence Scale (MMAS) and the EuroQoL 5D-3L (EQ-5D-3L) (both reported in 7/68 studies, 10% each). Fig 5 illustrates the tools used to assess each patient reported outcome and the scale of studies reporting on each tool.

#### Economic accessibility PREM

Seventeen out of 68 studies (25%) reported on an economic accessibility PREM. Economic accessibility was assessed primarily by measuring the lack of health insurance coverage, financial barriers to access services, and out-of-pocket expenditures leading to impoverishment.

### How were these PROMS used in practice?

While we did not find studies that evaluated how PROMs were being used in routine care, the authors proposed how their findings would influence the use of PROMs for clinical or policy decision-making. They recommended using PROMs to identify patients who did not meet treatment targets or who reported low treatment satisfaction [61, 107]. PROMs could be used to develop patient treatment plans including education to focus on improving clinical outcomes. They also promoted individualized treatment plans and patient-centered care where patients are involved in their treatment plans [24, 41, 48]. The PROMs contributed to the policymaking process by identifying gaps such as need for frequent screening of diabetes or hypertension, patient education programs, behavioral interventions, psychosocial support, task-shifting and other areas needing financial allocations [37, 39, 54, 61, 65, 67, 86, 95, 99, 107–113]. They can identify health system challenges leading to suboptimal care and barriers to achieving good outcomes that the policy makers can address such as cost of services, availability of medicines, waiting times, staff shortages, emergency response services [54, 95, 96, 114]. PROMs can also serve to assess implementation fidelity to clinical guidelines [115].

Some of the barriers noted by authors in the use of PROMs were related to the types of questions including the accuracy of self-reported measures [39, 41, 116], use of true/false dichotomies that do not capture the scale of response [107, 108], and lack of validated questionnaire translation [57, 61]. Some studies reported short follow-up periods that did not capture long-term clinical endpoints [99, 115].

## Discussion

To our knowledge, this scoping review is the first to shed light on the use of PROMs related to diabetes and hypertension in LMICs. We found that PROMs for diabetes and hypertension are being used in every region of the world, and more in upper-middle income countries than in low-income countries. Reported PROM use has increased over time.

An emphasis on improving healthcare quality, especially in the context of universal health coverage, may have raised the profile of PROMs. Increased attention to PROMs in high-income countries may translate to greater focus on PROMs in LMICs, particularly given the use of PROMs for clinical decision-making and policies.

PROMs have been successfully used to improve quality of care of chronic diseases in high-income countries. Mirroring this impact in LMICs will require appropriate contextual adaptation. PROMs should be validated in LMICs. Further work on translation to other languages would increase accessibility and applicability in LMICs.

PROMs can be used to create a feedback loop between providers and patients by identifying patient concerns and addressing system-level factors to improve health outcomes. At a facility level, a short, standardized validated questionnaire could be used as part of routine clinical practice to improve day-to-day patient care. PROMs data could be used locally and aggregated at subnational and national levels. In addition, PROMs can be included in national population-based surveys on diabetes and hypertension. At the regional and national levels, structured, regular PROMs assessment can track progress and inform benchmarking.

Simplifying use can facilitate increased adoption. Future research could focus on testing and validating short, simplified, and generic PROMs in LMICs that could be used across multiple NCDs and easily incorporated into routine health systems to allow for standardization and monitoring of trends as well as comparisons between individuals, health facilities, and across countries. In addition, a review of PROMs psychometric properties may be warranted to ensure that they are appropriate to the context.

Studies reported a diverse range of patient-reported outcomes. Most studies from low-income countries reported on health literacy and treatment adherence, while disease control and acute complications were the most common focus in middle-income countries. Disease control monitoring requires costly tests, such as HbA1c, which is more commonly measured in upper-middle-income countries. This contrasts with the reliance of many low-income countries on the more affordable approach of blood glucose measurement. Low-income countries may also need to address pressing needs related to infectious disease and maternal and child health, reducing emphasis on non-communicable diseases. Such constraints may lead low-income country health ministries to prioritize measurement of service access over disease outcomes, as this is more fully within health system control. Survival was not included as an outcome because included studies focused on patient-reported data. However, survival would be an outcome of interest for longitudinal studies as well as routine surveillance systems following PROMs over time.

High systolic blood pressure is estimated to be seven times more prevalent than diabetes in LMICs, yet most included studies focused on diabetes [117]. Chronic complications of diabetes can be overt, such as diabetic foot, retinopathy, and neuropathy. Diabetes care may be more variable and more intensive than hypertension care and diabetes patients may interact comparatively frequently with the health system. In contrast, at a population level, hypertension patients are more likely to be unaware of their condition, asymptomatic, or not on treatment. Given the global burden of high blood pressure and frequent co-morbidity between diabetes and hypertension, additional work is needed to collect data on PROMs on hypertension.

We identified only four studies that reported PROMs use for diabetes and hypertension comorbidity, based in Mexico (2), Brazil (1), and China (1). High comorbidity and disease burden may have motivated this interest in these three upper middle-income countries where hypertension and diabetes prevalence are above the respective global averages of 31% and 9.3% [3, 5]. Since PROMs are not routinely incorporated into health systems, most reported data required stand-alone surveys that require time, money, and human resources. Health systems may prefer to focus on health promotion and primary and secondary prevention of future complications based on laboratory-based evidence rather than patient-reported well-being of diagnosed patients [118]. Yet PROMs can incentivize value-based, person-centered care by providing feedback that can improve clinical care, change clinical pathways, and improve treatment outcomes, thereby responding to a particular need as many LMICs expand access through the rollout of universal health coverage.

Our study had several limitations. Although our search strategy was comprehensive, we may have omitted relevant publications not available in the English language or not indexed in PubMed, Embase or ClinicalTrials.gov. Limiting our study search to LMICs constrained our ability to comment on the scale and focus of PROMs in high-income countries. Authors were not consistent in reporting the type of tool used, modes of administration, version, content, and language, revealing variation in the quality of methodologies used. We therefore did not have sufficient data to provide the specific content measured by each tool in each instance of its use. It is possible that hypertension patient outcomes are being measured or described differently from the language used in this scoping review search strategy, leading to the absence of identified hypertension-specific tools. Our focus on patient-reported outcomes meant that we excluded studies that captured only clinical data. As a result, disease control as an outcome is reported only if it was reported alongside other relevant PROMs. It is therefore likely that disease control is measured more widely in LMICs than was captured in this review. Not all LMICs using PROMs may have published their practice in peer-reviewed journals. Finally, our study was limited to PROMs and one PREM related to economic access. We did not collect data on other PREMs, such as access to services. It is possible that low-income countries collect more data on affordability and geographical access than patient-reported outcomes in order to address service provision and patient experience.

## Conclusions

This scoping review provides a comprehensive overview of where, how, and what PROMs for diabetes and hypertension are being used in LMICs. PROMs are increasingly used all over the world, although less widely in low-income countries than in middle-income countries. Future research should address how PROMs can be incorporated into routine health systems while addressing various challenges, including inconsistencies in administration and specific patient-reported outcomes collected, paper-based data management systems, and resources for tool translation and validation. Development of a simple universal tool with a minimum of key elements that are reported by all patients could reduce costs, allow for incorporation into existing data systems, and facilitate cross-country and cross-condition comparisons. This ongoing tracking could be augmented by periodic in-depth surveys. PROMs provide an exciting opportunity to encourage person-centered, high-quality care.

## Supporting information

S1 Checklist PRISMA-ScR checklist. (DOCX)

S1 Appendix Full search strategy in PubMed format. (DOCX)
