# Supplementary material for: Patient-reported outcomes for diabetes and hypertension care in low- and middle-income countries: A scoping review
Source: PLoS One. 2021 Jan 15;16(1):e0245269. doi: 10.1371/journal.pone.0245269 (PMC7810280; doi:10.1371/journal.pone.0245269)
Supplement: S1 Appendix — (DOCX) [file pone.0245269.s002.docx]

**S1 Appendix Full search strategy in PubMed format**

(((patient reported outcome measures[mh] OR "patient reported" OR "patients reported" OR reported by patient* OR "patient view" OR "patients views" OR views of patient*) AND (diabetes[mh] OR diabetes[ti] OR diabetic*[ti] OR hypertension[mh] OR hypertens*[ti] OR high blood pressure*[ti]))

OR

((Patient outcome assessment[mh] OR "patient outcomes" OR "patient outcome" OR patient centered* OR promis[tiab]) AND (surveys and questionnaires[mh] OR survey* OR questionnaire*) AND ("patient satisfaction" OR "quality of life" OR "life quality" OR wellbeing OR survival[tiab] OR activities of daily living[mh] OR "activities of daily living" OR health services utilization OR depression[tw] OR patient acceptance of health care[mh] OR "patient acceptance" OR "medication adherence" OR "treatment adherence" OR "patient compliance") AND (diabetes[mh] OR diabetes[ti] OR hypertens*[ti] OR high blood pressure*[ti])))

AND

2009: 2019[pdat]

AND

English[la]

AND

(Afghanistan[tw] OR Albania[tw] OR Algeria[tw] OR Samoa[tw] OR Angola[tw] OR Antigua[tw] OR Barbuda[tw] OR Argentina[tw] OR Armenia[tw] OR Azerbaijan[tw] OR Bangladesh[tw] OR Belarus[tw] OR Belize[tw] OR Benin[tw] OR Bhutan[tw] OR Bolivia[tw] OR Bosnia[tw] OR Herzegovina[tw] OR Botswana[tw] OR Brazil[tw] OR Bulgaria[tw] OR Burkina Faso[tw] OR Burundi[tw] OR Cambodia[tw] OR Cameroon[tw] OR Cabo Verde[tw] OR Central African Republic[tw] OR Chad[tw] OR Chile[tw] OR China[tw] OR Colombia[tw] OR Comoros[tw] OR Congo[tw] OR Costa Rica[tw] OR Côte d'Ivoire[tw] OR Cote d’Ivoire[tw] OR Ivory[tw] OR Cuba[tw] OR Djibouti[tw] OR Dominica[tw] OR Dominican[tw] OR Ecuador[tw] OR Egypt[tw] OR Salvador[tw] OR Eritrea[tw] OR Ethiopia[tw] OR Fiji[tw] OR Gabon[tw] OR Gambia[tw] OR Georgia[tw] OR Ghana[tw] OR Grenada[tw] OR Guatemala[tw] OR Guinea[tw] OR Guinea-Bissau[tw] OR Guyana[tw] OR Haiti[tw] OR Honduras[tw] OR India[tw] OR Indonesia[tw] OR Iran[tw] OR Iraq[tw] OR Jamaica[tw] OR Jordan[tw] OR Kazakhstan[tw] OR Kenya[tw] OR Kiribati[tw] OR Korea [tw] OR Kosovo[tw] OR Kyrgyz [tw] OR Lao[tw] OR Laos[tw] OR Latvia[tw] OR Lebanon[tw] OR Lesotho[tw] OR Liberia[tw] OR Libya[tw] OR Lithuania[tw] OR Macedonia[tw] OR Madagascar[tw] OR Malawi[tw] OR Malaysia[tw] OR Maldives[tw] OR Mali[tw] OR Marshall[tw] OR Mauritania[tw] OR Mauritius[tw] OR Mexico[tw] OR Micronesia[tw] OR Moldova[tw] OR Mongolia[tw] OR Montenegro[tw] OR Morocco[tw] OR Mozambique[tw] OR Myanmar[tw] OR Namibia[tw] OR Nepal[tw] OR Nicaragua[tw] OR Niger[tw] OR Nigeria[tw] OR Pakistan[tw] OR Palau[tw] OR Panama[tw] OR Papua New Guinea[tw] OR Paraguay[tw] OR Peru[tw] OR Philippines[tw] OR Romania[tw] OR Russia[tw] OR Russian[tw] OR Rwanda[tw] OR Samoa[tw] OR Sao Tome[tw] OR Senegal[tw] OR Serbia[tw] OR Seychelles[tw] OR Sierra Leone[tw] OR Solomon Islands[tw] OR Somalia[tw] OR South Africa[tw] OR Sri Lanka[tw] OR St. Lucia[tw] OR St. Vincent[tw] OR Grenadines[tw] OR Sudan[tw] OR Suriname[tw] OR Swaziland[tw] OR Syrian[tw] OR Syria[tw] OR Tajikistan[tw] OR Tanzania[tw] OR Thailand[tw] OR Timor-Leste[tw] OR Togo[tw] OR Tonga[tw] OR Tunisia[tw] OR Turkey[tw] OR Turkmenistan[tw] OR Tuvalu[tw] OR Uganda[tw] OR Ukraine[tw] OR Uruguay[tw] OR Uzbekistan[tw] OR Vanuatu[tw] OR Venezuela[tw] OR Vietnam[tw] OR “West Bank”[tw] OR Gaza[tw] OR Yemen[tw] OR Zambia[tw] OR Zimbabwe [tw] OR developing countries[mh] OR “developing countries”[tw] OR “resource-limited”[tw] OR “resource-constrained”[tw] OR “low- and middle-income”[tw] OR LMIC[tw] OR “third world”[tw] OR “low income countries”[tw])
